# Involvement of the Glycogen Synthase Kinase-3 Signaling Pathway in TBI Pathology and Neurocognitive Outcome

**DOI:** 10.1371/journal.pone.0024648

**Published:** 2011-09-15

**Authors:** Pramod K. Dash, Daniel Johnson, Jordan Clark, Sara A. Orsi, Min Zhang, Jing Zhao, Raymond J. Grill, Anthony N. Moore, Shibani Pati

**Affiliations:** 1 Department of Neurobiology and Anatomy, The University of Texas Health Science Center at Houston, Houston, Texas, United States of America; 2 Department of Integrated Biology and Pharmacology, The University of Texas Health Science Center at Houston, Houston, Texas, United States of America; 3 Center for Translational Injury Research, The University of Texas Health Science Center at Houston, Houston, Texas, United States of America; Inserm U901, France

## Abstract

**Background:**

Traumatic brain injury (TBI) sets in motion cascades of biochemical changes that result in delayed cell death and altered neuronal architecture. Studies have demonstrated that inhibition of glycogen synthase kinase-3 (GSK-3) effectively reduces apoptosis following a number of stimuli. The Wnt family of proteins, and growth factors are two major factors that regulate GSK-3 activity. In the absence of stimuli, GSK-3 is constitutively active and is complexed with Axin, adenomatous polyposis coli (APC), and casein kinase Iα (CK1α) and phosphorylates ß-Catenin leading to its degradation. Binding of Wnt to Frizzled receptors causes the translocation of GSK-3 to the plasma membrane, where it phosphorylates and inactivates the Frizzled co-receptor lipoprotein-related protein 6 (LRP6). Furthermore, the translocation of GSK-3 reduces ß-Catenin phosphorylation and degradation, leading to ß-Catenin accumulation and gene expression. Growth factors activate Akt, which in turn inhibits GSK-3 activity by direct phosphorylation, leading to a reduction in apoptosis.

**Methodology/Principal Findings:**

Using a rodent model, we found that TBI caused a rapid, but transient, increase in LRP6 phosphorylation that is followed by a modest decrease in ß-Catenin phosphorylation. Phospho-GSK-3β immunoreactivity was found to increase three days post injury, a time point at which increased Akt activity following TBI has been observed. Lithium influences several neurochemical cascades, including inhibiting GSK-3. When the efficacy of daily lithium was assessed, reduced hippocampal neuronal cell loss and learning and memory improvements were observed. These influences were partially mimicked by administration of the GSK-3-selective inhibitor SB-216763, as this drug resulted in improved motor function, but only a modest improvement in memory retention and no overt neuroprotection.

**Conclusion/Significance:**

Taken together, our findings suggest that selective inhibition of GSK-3 may offer partial cognitive improvement. As a broad spectrum inhibitor of GSK-3, lithium offers neuroprotection and robust cognitive improvement, supporting its clinical testing as a treatment for TBI.

## Introduction

Both clinical and experimental studies have shown that the pathophysiology of traumatic brain injury (TBI) is complex and involves both primary and secondary injuries. Primary pathologies develop extremely rapidly after the traumatic event, and are typically not amenable to pharmacological interventions. In addition to causing immediate physical damage to the brain, the primary injury sets in motion cascades of molecular, cellular, genomic and metabolic processes that result in secondary injury [1], [2]. Secondary injury encompasses a number of pathological processes such as neuronal cell death and changes in neuronal architecture that can continue to evolve over time [3]–[6]. The hippocampus, a structure critically involved in learning and memory, is highly vulnerable to insults to the brain. Studies have shown that apoptotic loss of hippocampal neurons continues for days-to-weeks after the initial injury [7], [8], an effect thought to contribute to the learning and memory impairments observed following experimental TBI and in persons with brain trauma.

**Figure 1:**
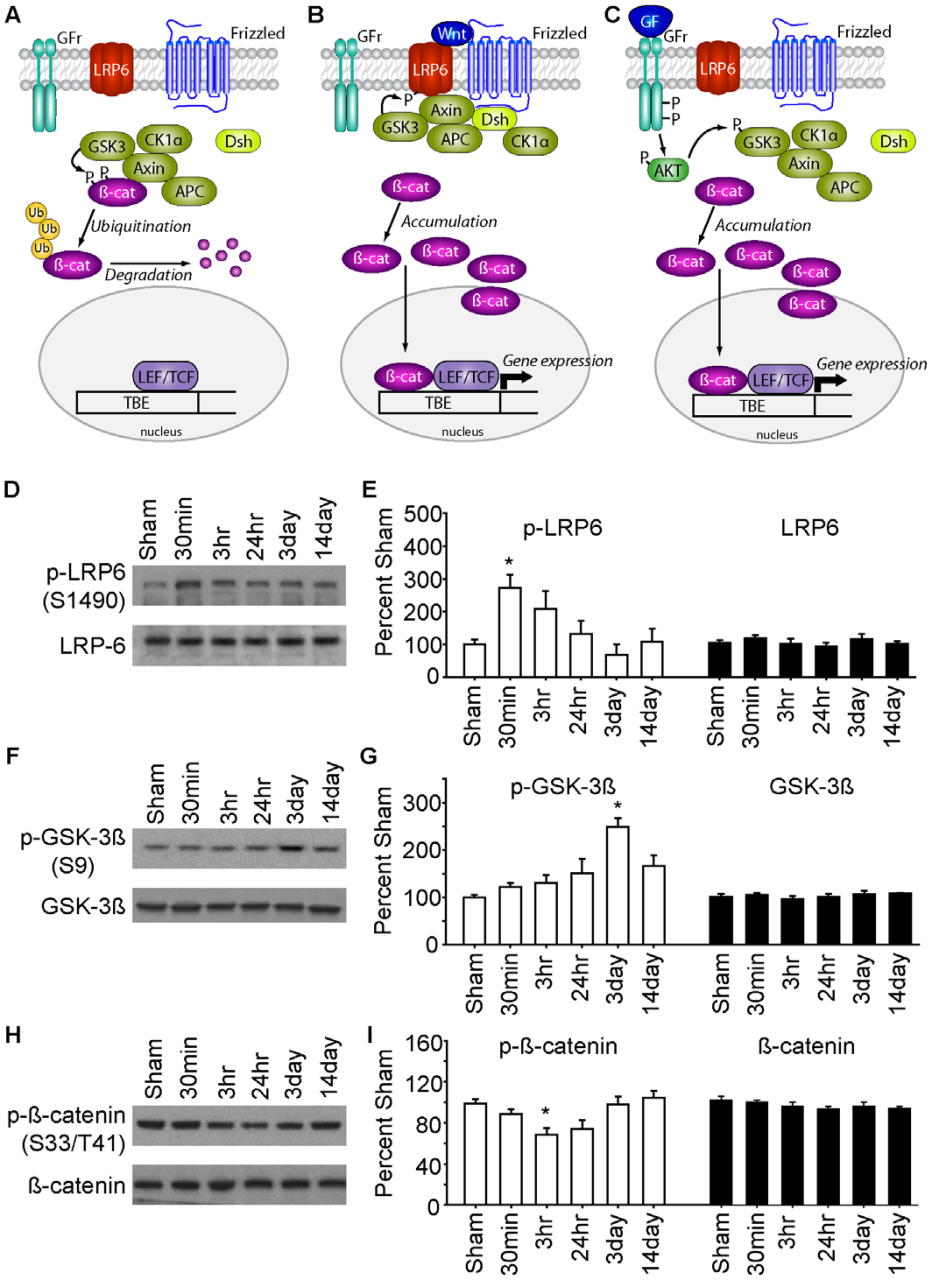
TBI alters the regulation of GSK-3. **A**) Schematic diagram showing the GSK-3 cascade. In the absence of Wnt signaling, GSK-3 complexes with APC, Axin, and casein kinase 1α (CK1a) and phosphorylates ß-Catenin (ß-cat) leading to its ubiquitination (Ub) and degradation. **B**) Upon Wnt binding to the Frizzled-LRP6 receptors, GSK-3 is translocated to the membrane via binding of Disheveled (Dsh) where it can phosphorylate LRP6, and allow for ß-Catenin accumulation and gene expression (via interaction with LEF/TCF transcription factors). **C**) Growth factor binding can result in Akt-mediated phosphorylation and inactivation of GSK-3. Rats (n = 5/time point) were subjected to TBI and hippocampi removed at various time points for western blot analysis. Representative images of western blots for **D**) phospho- and total LRP6, **F**) phospho- and total GSK-3ß, and **H**) phospho- and total ß-Catenin are shown. **E**) Summary data shows that TBI causes a transient increase in the phosphorylation of LRP6 on Ser^1490^. **G**) The phosphorylation of GSK-3ß on Ser^9^ is increased in a delayed manner, at a time point consistent with its phosphorylation by Akt. **I**) ß-Catenin phosphorylation was decreased at time points consistent with GSK-3 translocation to LRP6, but not as a result of GSK-3 phosphorylation. No change in ß-Catenin accumulation was observed. Data are presented as the mean ± SEM. *, P<0.05 by one-way ANOVA compared to sham animals.

Glycogen synthase kinase3 (GSK-3) was originally identified as a regulator of glycogen metabolism. Its role has been expanded to include regulation of protein synthesis, cell proliferation, cell differentiation, microtubule dynamics, cell motility and apoptosis [9]. GSK-3 activity has been linked to neuronal death triggered by a number of stimuli such as prion proteins, p53-induced apoptosis and amyloid-beta toxicity [10]–[12]. Consistent with a role of GSK-3 activity in apoptosis, selective small molecular inhibitors of GSK-3β protect cells in response to these and other pro-apoptotic stimuli. Wnt and Akt (also called protein kinase B) are two major signaling pathways that have been shown to regulate GSK-3 activity via distinct mechanisms [13]. In most cells, GSK-3 exists in a constitutively active form as a part of a protein complex consisting of APC (adenomatous polyposis coli), axin, and casein kinase Iα that phosphorylates β-Catenin leading to its degradation (Figure 1A). Binding of Wnt to the Frizzled-LRP6 (lipoprotein-related protein 6) receptor complex causes translocation of GSK-3 to the plasma membrane, away from its cytoplasmic substrates, where it binds to and phosphorylates LRP6 (Figure 1B) [14]. This leads to reduced phosphorylation of ß-Catenin and decreased proteosomal degradation. In addition, GSK-3 activity can be regulated by phosphorylation in response to growth factor binding via Akt (Figure 1C). This phosphorylation and inactivation of GSK-3 is thought to play a role in the anti-apoptotic properties of Akt activation [15]. Although increases in Akt activity have been observed following TBI, this appears to be insufficient to prevent TBI-induced apoptotic cell death. We therefore hypothesized that augmentation of GSK-3 inhibition post TBI would attenuate cell death and improve neurocognitive outcome in brain injured animals.

In the present study, we examined the consequences of TBI on GSK-3 activity and post-TBI administration of GSK-3 inhibitors on neurocognitive outcome. Treatment of injured animals with lithium, a multi-target drug that inhibits GSK-3, offered significant neuroprotection and improved learning and memory in brain injured animals. However, the GSK-3-selective small molecule inhibitor SB-216763 resulted in improved motor function, but only modest cognitive enhancement that occurred in the absence of visible neuroprotection.

## Materials and Methods

### Ethics Statement

All experimental procedures were approved by the Animal Care and Use Committee of The University of Texas Health Science Center at Houston (protocol# AWC-08-046),and were conducted in accordance with the recommendations provided in the *Guide for the Care and Use of Laboratory Animals*. Protocols were designed to minimize pain and discomfort during the injury procedure and recovery period.

### Materials

Male Sprague-Dawley rats (275–300 g) were purchased from Charles River Laboratories (Wilmington, MA). Lithium chloride and SB-216763 was purchased from Sigma Aldrich (St. Louis, MO) and Tocris Bioscience (Ellisville, MO), respectively. Antibodies to phospho ß-Catenin (Ser^33/37^), total ß-Catenin, phospho GSK-3β (Ser^9^), total GSK-3β, phospho-Erk1/2 (Thr^202^/Tyr^204^, Thr^185^/Tyr^187^), total Erk1/2antibodies, phospho-Akt (Ser^473^), total Akt, phospho-LRP6 (Ser^1490^), and total LRP6 were purchased from Cell Signaling Technology (Danvers, MA). Antibodies to NeuN were obtained from Millipore (Billerica, MA).

### Production of traumatic brain injury

All experimental procedures were approved by the Institutional Animal Care and Use Committee and were conducted in accordance with the recommendations provided in the *Guide for the Care and Use of Laboratory Animals*. Protocols were designed to minimize pain and discomfort during the injury procedure and recovery period. An electromagnet-driven controlled cortical impact (CCI) device was used to cause brain injury as previously described [16]–[18]. Briefly, animals were anesthetized using 5% isofluorane with a 1∶1 O_2_/N_2_O mixture, mounted a stereotaxic frame, and a midline incision was made. Bilateral 6-mm craniectomies were produced midway between the bregma and lambda with the medial edges of the craniectomies 1-mm lateral to the midline. Rats received a single impact (2.7 mm deformation) on the right parietal lobe with an impact velocity of 6 meters/sec. Core body temperature was maintained at 37-38°C by use of a heating pad. The animals were given time to recuperate in a warming chamber before being returned to their home cages. Animals were weighed daily after the injury for the first 3 days, then weekly thereafter.

### Drug preparation and administration

Lithium chloride was dissolved in sterile saline to a final concentration of 25 mg/ml. For testing the influence of lithium chloride on cognitive function following TBI, animals were injured then injected (s.cu.) with 0.5 ml (1 mEq/kg lithium) or an equal volume of vehicle 30 minutes post-injury, and every 24 hours thereafter for the first 5 days post-injury (n = 10/group). For biochemical analysis, naïve rats were given an s.cu. injection of lithium (0.1, 0.25, 0.5 or 1 mEq/kg) or vehicle once a day for 5 continuous days (n = 3/dose). Three hrs after the last injection, rats were killed by decapitation and hippocampi removed for the preparation of protein extracts. For single dose studies, 1.0 mEq/Kg lithium was injected, and the animals killed 3 hr later. The selective GSK-3β inhibitor, SB-216763, was dissolved in DMSO to a stock concentration of 8.33 mg/mL and further diluted in 60% PEG400 and 30% distilled water for a final concentration of 0.83 mg/mL. For testing the influence of GSK-3β inhibition on cognitive function following TBI, animals were i.p. injected with 5 mg/kg SB-216763 or an equal volume of vehicle 30 min following injury and every 24 hours thereafter for the first 5 days after injury (n = 10/group). For biochemical analysis, uninjured rats were given a single i.p. injection with SB-216763 (2.5 or 5 mg/kg) and killed 1 hr later (n = 3/dose).

### Sample Preparation

Hippocampal tissues were quickly removed while submerged in ice cold artificial cerebral spinal fluid (10 mM HEPES pH 7.2, 1.3 mM NaH_2_PO_4_, 3 mM KCl, 124 mM NaCl, 10 mM dextrose, 26 mM NaHCO_3_ and 2 mM MgCl_2_) containing phosphatase inhibitors (2 mM sodium fluoride, 2 mM sodium molybdate and 1 mM sodium orthovanadate). Tissues were homogenized (20 strokes) in 10 volumes of a buffer containing 10 mM Tris pH 7.4, 1 mM EGTA, 1 mM EDTA, 0.5 mM DTT, phosphatase inhibitors (0.1 µM okadaic acid and 1 mM sodium orthovanadate) and protease inhibitors (1 mM PMSF and 10 µg/ml leupeptin) using a motorized teflon-glass homogenizer. The samples were immediately aliquoted and frozen at −80°C.

### Western Blotting

Hippocampal tissue extracts were sonicated (5 pulses of 1 second each) using a Sonics Vibracell sonicator (Sonics & Materials, Inc., Newtown, CT) and a 0.4 mm diameter probe. The amount of protein in each sample was determined by a Bradford assay using bovine serum albumin (BSA) as the standard. Samples were denatured at 95°C for three minutes in 1x NuPage SDS sample buffer (Invitrogen, Carlsbad, Calif.). Equal amounts of protein were loaded, electrophoresed, and transferred to Immobilon-P membranes (Millipore, Billerica, MA) using the NOVEX X-Cell II system (Invitrogen, Burlingame, CA) and the buffers provided by the vendor. Membranes were blocked overnight in 5%BSA in TBST, followed by a three-hour incubation in primary antibodies (0.1–0.5 µg/ml in TBST containing 2% BSA) at room temperature. Membranes were then washed in TBST and incubated at room temperature with alkaline phosphatase (AP)-conjugated secondary antibodies for one hour as recommended by the vendor (Vector Laboratories, Burlingame, CA). Following extensive washing, immunoreactivity was detected using a chemiluminescence system and quantified using *Image J* (freely available through NIH).

### Assessment of motor function

All behavioral tests were conducted by an experimenter blind to the treatment groups. A vestibulomotor (beam balance) and a motor skill task (paw placement) were used to determine animals' motor performance on days 1–4 post-injury. For beam balance, rats were pre-assessed by placing them on a narrow wooden beam (1.5 cm wide) and measuring the duration they remained on the beam for up to 60 seconds. Animals were given repeated training until capable of balancing on the beam for the entire 60 sec period for 3 consecutive trials. Following injury, animals were given three daily trials during which the length of time spent on the beam was recorded. Paw placement was evaluated by placing the animal on a wire grid (opening size of 2×2 cm) and counting the number of foot faults out of a total of 50 steps. A foot fault was defined as when a front paw misses and appears below the plane of the grid. Paw placement was repeated three times to give an average daily score.

### Assessment of cognitive function

Rats were tested for their cognitive performance using the standard hidden platform version of the Morris water maze [17], [19]–[21]. All animals had recovered from the TBI-associated motor dysfunction prior to performing the cognitive testing. Animals were given 4 consecutive training trials per day with an inter-trial interval (iti) of 4min. If the animal failed to locate the platform within 60 sec on any given trial, it was led there by the experimenter. Thirty minutes after the last daily training trial, animals were given a probe trial to measure quadrant preference and platform localization. Twenty-four hours following the last day of training, animals were again tested in a probe trial to measure quadrant preference and platform localization. Movement within the maze was monitored using a video camera linked to tracking software (Ethovision, Noldus Information Technology, Leesbury, VA, USA).

### Contusion volume measurement

Following the completion of the behavioral studies, approximately 28 days following the injury, animals (n = 10/group) were deeply anesthetized with sodium pentobarbital (100 mg/kg) and transcardially perfused with phosphate buffered saline (PBS) followed by 4% paraformaldehyde. Brains were removed, post-fixed overnight in perfusant, then cryoprotected in a 30% sucrose solution. Cortical tissue loss was estimated essentially as described previously [22], by experimenters kept blind with respect to the treatment groups. In brief, cryosections (40 µm thickness) spanning the rostral-caudal extent of the injured cortex were selected and stained with cresyl violet by an experimenter given only the animal's identifier code. Images of the resultant slides were then used for tissue loss measurement by a second experimenter. The area of cortical tissue loss for each section was carefully outlined using *Adobe Illustrator*, with the area of the resultant outlines quantified by *Image J*, from the National Institutes of Health. Contusion volume was calculated using the equation A_1_(0.5X_1_)+A_2_(0.5X_1_ +0.5X_2_)+A_n-1_(0.5X_n-1_ +0.5X_n_)+A_n_(0.5X_n_) where A is the area (mm^2^) of the contusion for each slice, and X is the distance (mm) between two sequential slices. Once the contusion volume had been calculated for each animal, the blind code was broken and group differences assessed.

### Immunohistochemistry

For post-TBI histological evaluation, tissue sections generated for the measurement of cortical contusion volume were used for immunohistochemistry. Free-floating slices were incubated overnight in primary antibody (0.5–1.0 µg/ml) in TBS containing 2% BSA and 2.5% normal goat serum. After extensive washing, immunoreactivity was detected using species-specific secondary antibodies coupled to Alexafluors. These fluorescently-labeled tissue sections were evaluated by a blind observer to identify any potential differences between the treatment groups. As visible neuroprotection was detected following lithium administration, stereological cell counts were carried out. For cell counts, NeuN immunostaining was performed using a colorimetric detection system consisting of a secondary antibody conjugated to horseradish-peroxidase and diaminobenzadine (DAB) as the chromagen. This was done to avoid the photobleaching often associated with high magnification imaging of fluorescent immunohistochemistry.

### Stereology

A blind counting methodology was employed for determination of NeuN positive cells in the CA3 layer of the ipsilateral hippocampus in lithium chloride- and vehicle-treated animals (n = 5/group). Every tenth section through the ipsilateral dorsal hippocampus was processed for NeuN immunoreactivity using colorimetric detection as described above. NeuN positive cells within the CA3 subfield were counted using the optical dissector technique using *Stereo Investigator* (MicroBrightField Bioscience, Williston, VT) [23]. The CA3 subfield was differentiated from the CA2 by the density of cells within these layers. As there is no morphological distinction between the CA3 and CA4 subfields using NeuN staining, these subfields were divided by dropping a vertical line from the lateral edge of the inner blade of the dentate gyrus. Cells in the outermost planes of focus (5 µm) were omitted to avoid counting cell caps. The number of NeuN-labeled cells in approximately twenty, computer chosen areas within the CA3 cell layer was scored for each section. The counting frame was 108×108 µm. The size of the counting frame, and the number of grid sections was determined based on preliminary cell counts. The number of NeuN labeled cells/mm^3^ for each section was obtained from the estimated cells divided by the contour volume. The number of cells/mm^3^ for each animal was calculated as the average of the number of cells/mm^3^ from each section examined.

### Statistical Analysis

For evaluation of behavioral data, repeated measures analyses of variance (two-way or one-way as appropriate) and t-tests were utilized to determine statistical differences. A Holm-Sidak method for multiple comparisons post-hoc test was used to determine data points with significant differences. Dose response western data were evaluated using a one-way ANOVA followed by a Dunnet's *post-hoc* test. For data that did not pass a Shapiro-Wilk normality test, appropriate non-parametric analysis was performed. Data were considered significant at p≤0.05 and presented as Mean ± Standard Error of the Mean (S.E.M.).

## Results

### TBI causes transient inhibition of the GSK-3 pathway

To determine the influence of TBI on GSK-3 activity, western blots were performed to examine the phosphorylation of LRP6, GSK-3ß, and ß-Catenin in hippocampal extracts prepared at various time points following injury (n = 5/time point). Figure 1D shows images of representative westerns illustrating the phosphorylation of the LRP6 receptor on Ser^1490^. This site has been shown to be phosphorylated in response to GSK-3 translocation to the LRP6 signalosome [14]. The summary data presented in Figure 1E show that the phosphorylation of LRP6 is significantly increased by 30 min post-injury (one-way ANOVA: F_(5,24)_ = 4.297; P = 0.006). The total levels of LRP6 did not significantly change at any of the time points examined (one-way ANOVA: F_(5,24)_ = 0.587; P = 0.710). As described above, GSK-3 is also inhibited following phosphorylation by Akt. Previous studies have reported that cortical impact injury results in increased Akt phosphorylation (on Thr^308^) and activity 3-day post-injury [24]. We therefore examined if GSK-3 phosphorylation is enhanced 3-day post-CCI. The representative western blots and the summary data shown in Figures 1F and 1G demonstrate that the phosphorylation of Ser^9^ of GSK-3ß increases after injury, reaching significance by the 3-day time point (one-way ANOVA: pGSK-3 F_(5,24)_ = 7.335; p<0.001). This is consistent with a previous study which showed that GSK-3 phosphorylation is increased in the hippocampus for at least 24 hr after a weight drop model of TBI [25]. No change in the total levels of GSK-3ß was detected (Figure 1G; one-way ANOVA: GSK-3 F_(5,24)_ = 0.622; p = 0.684).

Upon inactivation by either the Wnt-LRP6 or Akt cascades, GSK-3-mediated phosphorylation of ß-Catenin on Ser^33/37^ is reduced, leading to its nuclear accumulation and enhanced gene expression. To assess if the TBI-associated decreases in GSK-3 activity are associated with changes in ß-Catenin phosphorylation and levels, western blots were again carried out. Figure 1H shows representative western blots illustrating phospho-ß-Catenin immunoreactivity at different time points following injury. The summary data in Figure 1I demonstrates that TBI causes a significant, but transient, decrease in the phosphorylation of Ser^33/37^ of ß-Catenin acutely following injury (one-way ANOVA: F_(5,24)_ = 4.948; p = 0.003). Although a significant decrease in ß-Catenin phosphorylation was observed by 3 hr post-injury, no concurrent increase in the total levels of this protein was observed (one-way ANOVA: F_(5,24)_ = 1.208; p = 0.336). No changes in either the phosphorylation or total levels of ß-Catenin were observed at the time point at which GSK-3ß phosphorylation was observed.

### Lithium treatment improves hippocampal function and reduces neuronal loss following TBI

**Figure 2:**
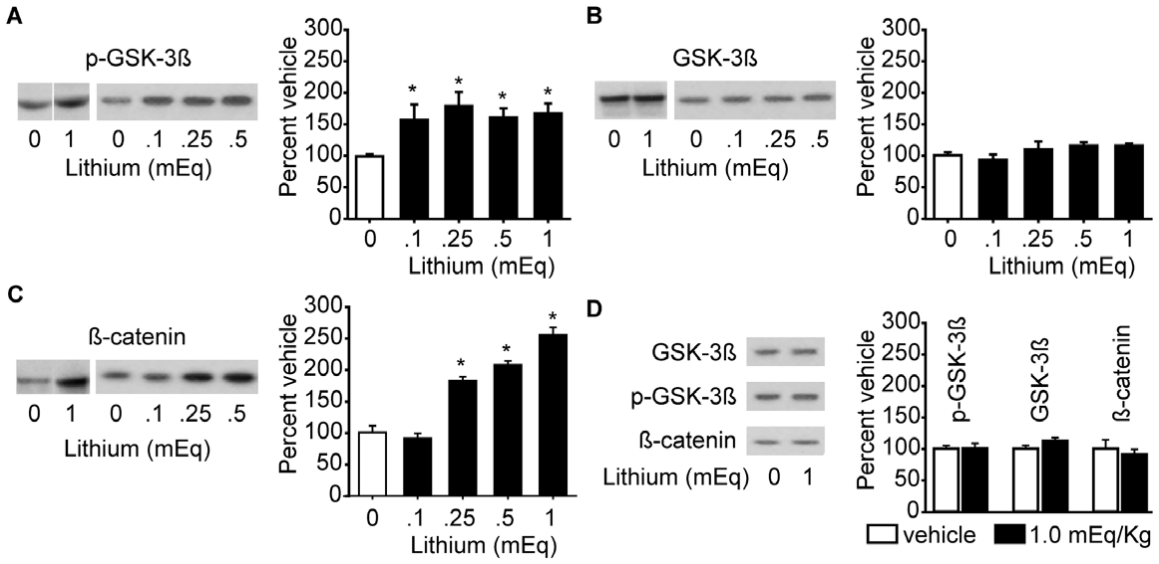
Systemic lithium administration inhibits hippocampal GSK-3 activity. **A**) Representative western blots showing the dose response of different amounts of lithium (injected daily for 5 days) on GSK-3ß phosphorylation. The summary data shows that all doses of lithium were found to increase GSK-3 phosphorylation. **B**) Representative western blots and summary data showing that the different doses of lithium had no effect on the total levels of GSK-3ß. **C**) Representative western blots and summary data showing that lithium administration causes a significant accumulation of ß-Catenin, with maximal levels seen at the 1.0 mEq dose. **D**) Representative western blots and summary data showing that a single dose of 1.0 mEq/Kg lithium has no effect on the phosphorylation or total levels of GSK-3ß, nor on ß-Catenin accumulation. Data are presented as the mean ± SEM. *, P≤0.05 by one-way ANOVA compared to vehicle (0 dose) injected animals. n = 3/group

Inhibition of GSK-3 has been shown to reduce neuronal death triggered by a number of stimuli [13]. Although GSK-3 activity appears to be modulated by both receptor- and Akt-mediated mechanisms following brain injury, these effects were transient and apparently insufficient to prevent TBI-associated hippocampal cell death. In order to determine the consequences of further inhibition of the GSK-3 pathway, post-injury administration of lithium was tested. A number of previous studies have shown that many of the protective effects of lithium are mediated by its influence on the GSK-3 cascade [26], although several other targets of its action have been reported. To confirm that systemic administration of lithium can inhibit the GSK-3 cascade in the brain, animals were s.cu. injected with 1 mEq/kg lithium (n = 3) or saline (n = 5) for five consecutive days. This dose was chosen based on a previous study that demonstrated that delayed administration of 1mEq lithium after cerebral ischemia reduced cortical infarct volume and facilitated neurological recovery [27]. In addition, we also tested the influence of lower doses of lithium, namely 0.10, 0.25, and 0.50 mEq/Kg (n = 3/dose). Three hours following the last injection, hippocampal protein extracts were prepared for western blots. Figure 2A shows representative western blots of phosphorylated (Ser9) GSK-3ß following different concentrations of lithium. Since the evaluation of the 1.0 mEq/Kg dose, and the dose-response study (0.1, 0.25, and 0.5 mEq/Kg) were carried out at two different times, an independent vehicle group was used for each analysis. The summary data show that when normalized across experiments, all doses tested effectively increased the phosphorylation of GSK-3ß (one–way ANOVA: F_(4,12)_ = 5.326, p = 0.011). No significant difference was detected in the total levels of GSK-3ß (one–way ANOVA: F_(4,12)_ = 1.770, p = 0.200) (Figure 2B). Although all doses increased GSK-3 phosphorylation, significant accumulation of ß-Catenin was only observed at the high doses (one-way ANOVA: F_(4,12)_ = 49.283, p<0.001), with maximal accumulation observed at 1.0 mEq lithium (Figure 2C). Since maximal ß-Catenin accumulation was observed after 5 daily injections of the 1.0 mEq dose, we questioned if a single injection was sufficient to elicit this effect. To test this, animals received a single injection of either 1.0 mEq/Kg lithium or an equal volume of vehicle and killed 3 hr later. The western blot results shown in Figure 2D demonstrate that a single dose of lithium had no significant effect on GSK-3 phosphorylation (Student's *t-test*: 0.956), total GSK-3 levels (Student's *t-test*: 0.130) or ß-Catenin levels (Student's *t-test*: 0.587). Based on these results, we tested the consequences of daily post-injury administration of 1.0 mEq lithium for the first 5 days after injury using a series of behavioral and cognitive tasks.

**Figure 3:**
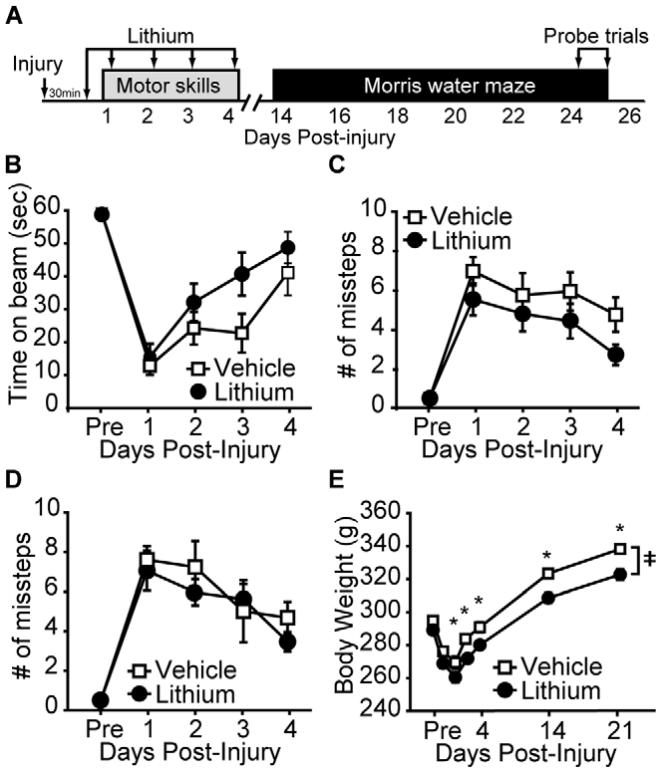
Post-TBI lithium administration has no effect on motor function, but exacerbates post-injury weight loss. **A**) Schematic of the injection and behavioral testing paradigm. Injured rats receiving 1 mEq/kg lithium (n = 10) performed similarly to vehicle-injected animals (n = 10) when tested for their **B**) balance beam performance, and **C**) ipsilateral and **D**) contralateral foot faults. **E**) Lithium exacerbated post-injury weight loss. Data are presented as the mean ± SEM. ≠, significant difference by repeated measures two-way ANOVA. *, P≤0.05.

The influence of post-injury lithium injection on vestibulomotor and motor function was tested on days 1–4 post-injury using the beam balance and foot fault tasks, respectively (Figure 3A). Rats were injured, and then injected s.cu. with 1mEq/kg (n = 10) or vehicle (n = 10) 30 min after the injury. Drug administration was continued every 24 hr for the first 5 days of injury. Figure 3B shows that post-injury administration of lithium did not significantly improve beam balance performance compared to simultaneously tested vehicle controls (repeated measures two-way ANOVA: F_(1,18)_ = 3.267, p = 0.087). In addition, neither ipsilateral (repeated measures two-way ANOVA: F_(1,18)_ = 2.033, p = 0.170; Figure 3C) nor contralateral (repeated measures two-way ANOVA: F_(1,18)_ = 1.589, p = 0.222; Figure 3D) foot faults were significantly different between the two groups. At the dose tested, however, lithium significantly exacerbated post-injury weight loss (two-way repeated measures ANOVA: F_(1,18)_ = 14.324, p = 0.001). All animals had normal vestibulomotor and motor function by day 7 post-injury (data not shown), and had returned to their pre-injury weight.

**Figure 4:**
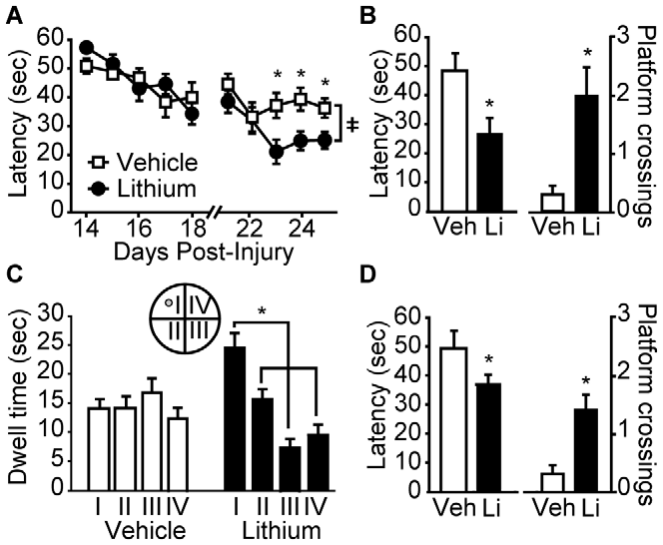
Post-injury administration of lithium chloride improves cognitive function following TBI. Rats treated with 1 mEq/kg lithium (n = 10) had improved spatial learning and memory as indicated by **A**) decreased latencies to the platform during training (≠, significant difference by repeated measures two-way ANOVA), and **B**) decreased latency to the first platform crossing and increased number of platform crossings during a probe trial given 24 hr after training (*, p≤0.05 by Student's t-test). **C**) Animals receiving lithium spent significantly longer time in the target quadrant (I) compared to the other quadrants (II, III, IV), suggesting improved localization (*, p≤0.05 by repeated measures one-way ANOVA). **D**) A probe trial given 30 min after training showed that the lithium-infused animals also had significantly shorter latencies and more crossings of the platform location compared to vehicle-infused animals (*, p≤0.05 by Student's t-test). Veh: vehicle; Li: lithium. Data are presented as the mean ± SEM.

When these animals were tested for their ability to perform the Morris water maze task (beginning on day 14 post-injury), the rats treated with lithium had learning curves that were significantly different from their vehicle-injected counterparts (significant interaction by repeated measures two-way ANOVA: F_(9,162)_ = 4.082, p<0.001; Figure 4A). When tested in a probe trial 24 hr following training, the lithium-treated injured animals crossed the original platform location significantly faster (vehicle: 48.52±6.17 sec; lithium chloride: 26.57±5.67 sec, p = 0.017) and more often (vehicle: 0.30±0.15 crosses; lithium chloride: 2.00±0.49 crosses, p = 0.003) than vehicle-infused animals, suggesting an improved memory for the platform location (Figure 4B). In support of this conclusion, the lithium chloride-injected rats demonstrated a preference for the quadrant in which the platform was located (repeated measures one-way ANOVA: F_(3,27)_ = 11.50, p<0.001) while the saline-injected controls did not (repeated measures one-way ANOVA: F_(3,27)_ = 0.62, p = 0.619) (Figure 4C). Comparison across groups revealed a significant interaction between treatment and quadrant (repeated measures two-way ANOVA: F_(3,54)_ = 6.58, p<0.001) with a significant difference in the time each group spent searching in the target quadrant (quadrant I). Short-term memory was found to be improved in animals treated with lithium, as evidenced by reduced latency to cross the original platform location (Student's *t-test* p = 0.034) and more number of platform crossings (Student's *t-test* p = 0.004) (Figure 4D) in a probe trial given 30 min post-training.

**Figure 5:**
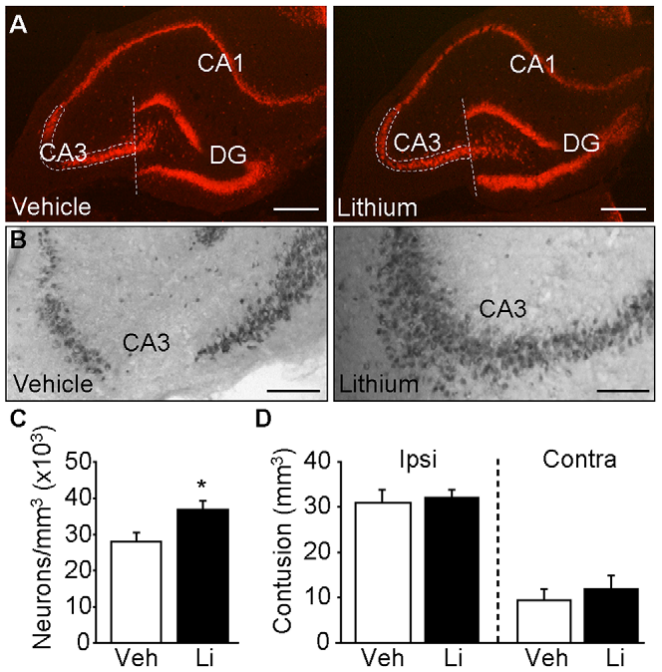
Post-injury administration of lithium reduces hippocampal neuronal cell loss. **A**) Representative photomicrographs of NeuN immunoreactivity from the dorsal, ipsilateral hippocampi of a vehicle- and a lithium-treated animal. Scale bar  = 500 µm. DG: dentate gyrus. The outlined area indicates the field in which the cell counts were carried out. **B**) High-magnification image showing NeuN immunoreactivity within the CA3 subfield from a vehicle- and a lithium-injected, injured animal. Scale bar  = 200 µm. Lithium treatment **C**) significantly reduced CA3 neuronal loss, but not **D**) cortical contusion volume after TBI. *, p≤0.05 by Student's t-test. Veh: vehicle; Li: lithium. Data are presented as the mean ± SEM.

The improved water maze performance we observed as a result of lithium treatment suggests that this drug may offer neuronal protection to hippocampal neurons after TBI. When the brains of the animals used for behavioral testing were examined for hippocampal neuron loss, an apparent preservation of ipsilateral CA3 pyramidal neurons (Figures 5A) was observed. To quantify the number of ipsilateral CA3 neurons, sections spanning the dorsal hippocampus from representative vehicle (n = 5)- and lithium (n = 5)-treated animals were immunoreacted for NeuN. Immunoreactivity was detected using a horseradish peroxidase-coupled secondary antibody, and then visualized using diaminobenzidine (Figure 5B). Stereological cell counts of CA3 pyramidal neurons were performed using the optical dissector probe of *Stereo Investigator*. These counts revealed that animals treated with lithium had significantly more ipsilateral CA3 pyramidal neurons than those counted in vehicle-injected animals (Figure 5C). Cortical contusion volume, however, was not significantly different between the two groups (Figure 5D).

### The GSK-3 inhibitor SB-216763 improves motor function following TBI

**Figure 6:**
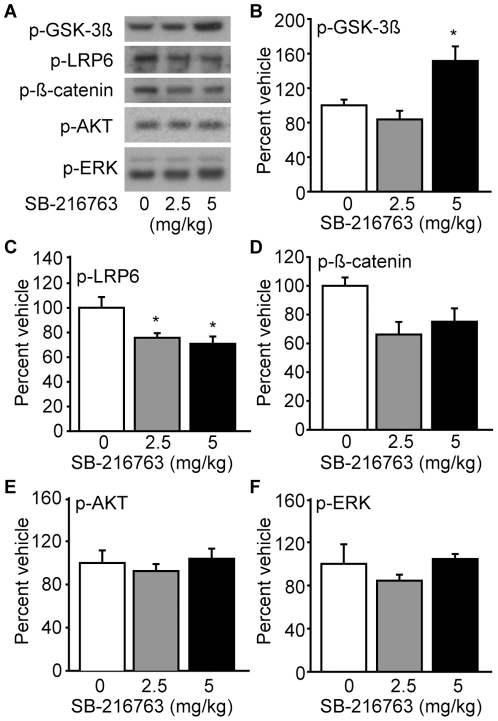
SB-216763 inhibits the GSK-3 pathway. **A**) Representative images of western blots for phospho-GSK-3ß, phospho-LRP6, phospho-ß-Catenin, phospho-Akt, and phospho-ERK in response to i.p. injection of SB-216763 (2.5 and 5.0 mg/kg) or vehicle (0 mg/kg) (n = 3/group). **B**) SB-216763 significantly increased the phosphorylation of GSK-3ß in a dose-dependent manner. The phosphorylation of the GSK-3 substrates **C**) LRP-6 and **D**) ß-Catenin, were found to be decreased following SB-216763 administration. The phosphorylation levels of **E**) Akt and **F**) ERK were unaffected by SB-216763 administration. Data are presented as the mean ± SEM. *, P<0.05 by one-way ANOVA.

As our results with lithium suggested that GSK-3 inhibition may offer neuroprotection, we next tested if a GSK-3-specific inhibitor could mimic the influences of lithium. SB-216763 has been shown to be a potent inhibitor of GSK-3, while having no significant influence on the activity of other kinases [26]. To test the efficacy of the drug, rats were i.p. injected with either 2.5 or 5.0 mg/kg SB-216763 (n = 3/group), euthanized 1 hr later, and the hippocampus dissected for the preparation of protein extracts. These doses were chosen based on previous studies indicating that SB-216763 can reduce D1 agonist-induced hyperactivity [28]. Representative western blots are shown in Figure 6A. Consistent with previous reports, Figure 6B shows that SB-216763 increases the phosphorylation of GSK-3*β* on Ser^9^ (one-way ANOVA: F_(2,6)_ = 8.833, p = 0.016), suggesting decreased GSK-3 activity. Associated with this was a significant decrease in the phosphorylation of LRP6 on Ser^1490^ (one-way ANOVA: F_(2,6)_ = 5.891, p = 0.038; Figure 6C) and a trend towards decreased ß-Catenin phosphorylation on Ser^33/37^ (one-way ANOVA: F_(2,6)_ = 4.697, p = 0.059; Figure 2D). No significant influence of SB-216763 was observed on the phosphorylation/activity of Akt (one-way ANOVA: F_(2,6)_ = 0.381, p = 0.698; Figure 6E) or ERK1/2 (one-way ANOVA: F_(2,6)_ = 0.865, p = 0.468; Figure 2F), nor on the total levels of any of the targets interrogated.

**Figure 7:**
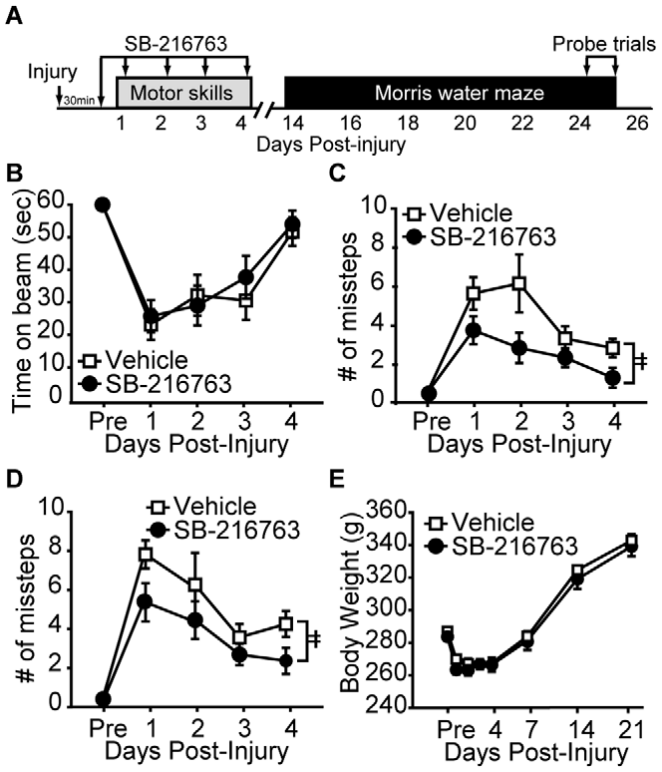
Post-injury administration of SB-216763 improves motor function. **A**) Schematic of the drug injection and behavioral testing paradigm. Rats (n = 10/group) were injected (i.p.) for the first 5 days post-injury with either 5.0 mg/kg SB-216763 or vehicle. SB-216763-treated rats had **B**) comparable vestibulomotor deficits, but made significantly fewer **C**) ipsilateral and **D**) contralateral foot faults. **E**) Body weight was unaffected by SB-216763 administration. Data are presented as the mean ± SEM. ‡, P<0.05 by two-way repeated measures ANOVA.

In order to determine the influence of SB-216763 on TBI outcome, rats were injured, then i.p. injected 30 minutes later with either 5.0 mg/kg SB-216763 or vehicle (n = 10/group). Rats continued to receive daily injections for the first 5 days after injury during which time they were tested for their vestibulomotor and motor performances (Figure 7A). Drug administration was continued for 5 days since previous studies have reported that apoptosis continues for days after TBI [29]. While SB-216763 injected animals did not perform any differently on the balance beam task compared to vehicle-injected control animals (repeated measures two-way ANOVA: F_(1,18)_ = 0.039, p = 0.846 (Figure 7B), they did show significantly less ipsilateral (Figure 7C; repeated measures two-way ANOVA: F_(1,18)_ = 4.74, p = 0.040) and contralateral (Figure 3D; repeated measures two-way ANOVA: F_(1,18)_ = 9.97, p = 0.004) foot faults as compared to their vehicle-injected counterparts. SB-216763 treatment did not have any significant influence on post-injury weight loss (Figure 7E; repeated measures two-way ANOVA: F_(1,18)_ = 0.282, p = 0.602). All animals had normal vestibulomotor and motor function by day 7 post-injury (data not shown).

**Figure 8:**
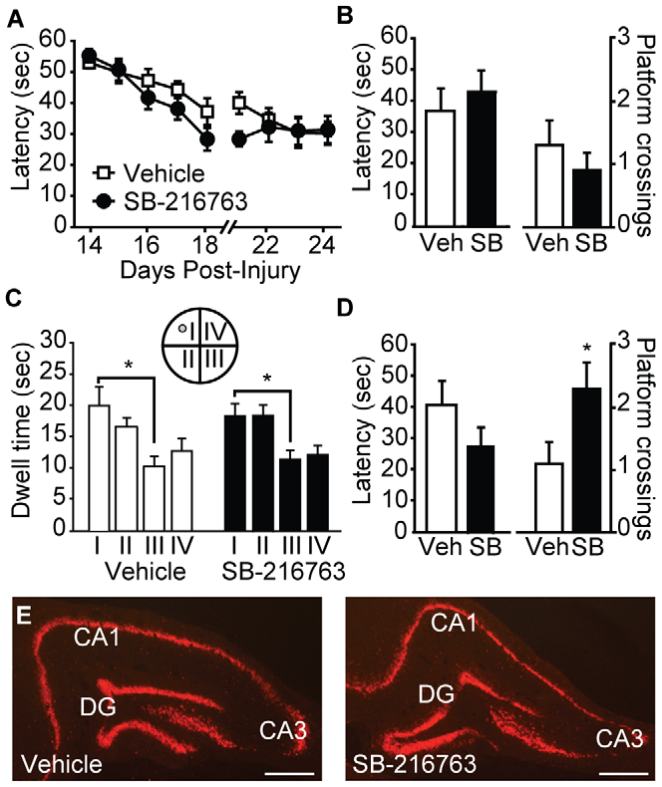
Post-injury administration of SB-216763 partially improves spatial memory, but does not offer neuroprotection. **A**) SB-216763-treated rats performed similarly to rats treated with vehicle in the hidden platform version of the Morris water maze task. No significant influence of SB-216763 administration was seen in either the **B**) latency to the first platform crossing or number of platform crossings, nor **C**) quadrant preference in a probe trial given 24 hr following training. *, P<0.05 by one-way repeated measures ANOVA. **D**) When given a probe trial 30 min after training, SB-216763-treated rats crossed the hidden platform location significantly more times than did the vehicle treated controls. *, P<0.05 by Student's t-test. **E**) Representative photomicrographs of NeuN immunoreactivity from the dorsal, ipsilateral hippocampi of a vehicle- and a SB-216763-treated animal. Data are presented as the mean ± SEM. DG: dentate gyrus. Scale bar  = 500 µm.

When evaluated for their performance in the standard hidden platform version of the Morris water maze task (beginning 14 days post-injury), no difference in learning was detected between injured rats treated with 5.0 mg/kg SB-216763 and those treated with vehicle (Figure 8A; repeated measures two-way ANOVA: F_(1,18)_ = 1.639, p = 0.217). Similarly, when a probe trial was given 24 hr after the completion of training, no differences were seen in the latency to cross (vehicle: 36.68±7.24 sec; SB-216763: 42.81±6.88 sec, p = 0.725) or the number of platform crossings (vehicle: 1.30±0.40 crosses; SB-216763: 0.90±0.28 crosses, p = 0.50) between the two groups (Figure 8B). Figure 8C shows that while both the vehicle (one-way repeated measures ANOVA: F_(3,27)_ = 3.186, p = 0.040) and SB-216763 (one-way repeated measures ANOVA: F_(3,27)_ = 4.019, p = 0.017) treated groups displayed significant preference for the target quadrant (I) relative to the starting quadrant (III), there was no significant difference between the two groups in their quadrant preferences (two-way repeated measures ANOVA: F_(1,18)_ = 0.971, p = 0.338). Interestingly, when a short term memory test was given at 30 minutes following training, SB-216763 injected animals crossed the platform location significantly more than the vehicle-injected controls (vehicle: 1.10±0.35 crosses;SB-216763: 2.30±0.42 crosses, p = 0.042; Figure 8D). No difference in latency (Figure 8D) nor quadrant preference (data not shown) was observed between the two groups during the short-term memory probe trial. Consistent with the lack of improvement in the water maze, histological examination did not reveal any overt differences in ipsilateral hippocampal neuronal loss (as indicated using NeuN immunoreactivity) between injured animals treated with vehicle and those given SB-216763 (Figure 8E), with both groups having demonstrable neuron loss in the dentate gyrus (DG) and CA3 subfields. Neither ipsilateral (vehicle: 28.30±2.98 mm^3^ vs. SB-216763: 28.29±2.98 mm^3^; Student's *t-test* p = 0.99) nor contralateral (vehicle: 14.48±2.81 mm^3^ vs. SB-216763: 12.39±2.61 mm^3^; Student's *t-test* p = 0.59) cortical tissue loss was different between the two groups.

## Discussion

Using a rodent model of TBI, we examined if post-injury inhibition of GSK-3 activity is neuroprotective, and if this neuroprotection is associated with improved motor and cognitive outcomes. Our findings show that daily, post-injury administration of the GSK-3 inhibitor lithium for five days significantly improved hippocampal-dependent learning and memory and reduced hippocampal CA3 neuron loss. In contrast, the GSK-3-selective inhibitor SB-216763 improved motor function, but did not provide overt neuroprotection and only modestly improved learning and memory.

One of the most studied mechanisms of GSK-3 regulation is the canonical Wnt pathway. In the absence of Wnt, GSK-3 exists as a part of a destruction complex consisting of the scaffold protein Axin, the tumor suppressor APC, and casein kinase Iα (CKIα) that binds to, and regulates, ß-Catenin-mediated gene expression [9] (see Figure 1A). Phosphorylation of ß-Catenin by CKIα and GSK-3 marks it for proteosomal degradation. Upon Wnt binding to the Frizzled receptor, GSK-3 is recruited to the plasma membrane where it can phosphorylate the Frizzled co-receptor LRP6 on five PPPSP motifs. These phosphorylation events prime the receptor for subsequent phosphorylation by CKIγ [30]. Several studies have indicated that the phosphorylation of LRP6 is crucial for reducing ß-Catenin phosphorylation and degradation [14]. When the phosphorylation of LRP6 was examined in hippocampal extracts following TBI, we observed a transient, but significant increase in Ser^1490^ phosphorylation, suggesting GSK-3 translocation. However, as other kinases have also been shown to phosphorylate LRP6 on Ser^1490^, we cannot rule out their involvement in the increased phosphorylation we observed [14]. Regardless of the mechanism underlying its phosphorylation, it appears that phosphorylated LRP6 acts, at least in part, to inhibit GSK-3. Using phosphorylated peptide mimetic of LRP6, it has been shown that peptides containing the PPPSPxS motif of LRP6 strongly inhibit GSK-3 [31], [32]. Consistent with increased Wnt signaling after TBI, the enhanced LRP6 phosphorylation we observed was followed by a significant decrease in GSK-3-dependent ß-Catenin phosphorylation.

In addition to Wnt signaling, GSK-3 has been shown to be inhibited by direct phosphorylation by a number of kinases including protein kinase A (PKA) [33], Akt [34], PKC [35], p90 ribosomal S6 kinase (p90RSK) [36] and p70 ribosomal S6 kinase (p70S6K) [37]. Our results indicate that TBI causes a delayed increase in GSK-3ß phosphorylation on Ser^9^. Although the kinase responsible for this phosphorylation has not been identified, the time point at which we observed the increase (3 days post-injury), is consistent with that seen for activation of Akt. Using a cortical impact model of injury, Zhang et al., reported that TBI causes a delayed phosphorylation of Akt on Thr^308^ that occurs 72 hr post-injury [38]. Although we observed a significant increase in GSK-3ß phosphorylation, no corresponding decrease in ß-Catenin phosphorylation was seen at this time point. While the reason for this is not clear at present, it is possible that the activity of GSK-3α, which can also phosphorylate ß-Catenin, was unaffected by TBI [39] and could have maintained ß-Catenin phosphorylation.

Accumulating evidence suggests that GSK-3 may play an important role in apoptosis with its overexpression inducing, and its inhibition preventing, apoptosis [9], [40], [41]. Although we observed that GSK-3 is negatively regulated as a result of TBI, the continued presence of neuron death suggests that this inhibition is not sufficient to protect the hippocampus from TBI-associated apoptosis [7], [8]. As several studies have indicated that administration of the GSK-3-selective inhibitor SB-216763 can protect cultured neurons against excitotoxicity and toxin-induced apoptosis [42]–[44], and it has been shown to reduce the presence of pyknotic neurons in a model of Alzheimer's disease [12], we questioned if similar benefits could be observed following TBI. In contrast to our anticipation, animals treated with the selective GSK-3 inhibitor SB-216763 had comparable hippocampal cell loss to injured rats treated with vehicle, and only showed a modest improvement in short-term memory. Our biochemical assessment of the consequences of SB-216763 demonstrated that the dose employed in our behavioral studies (5.0 mg/kg) effectively reduced GSK-3 activity in the hippocampus, and this dose has been used by other investigators to examine the consequences of GSK-3 inhibition in vivo [28]. While these results suggest that SB-216763 is only partially effective at reducing TBI pathology, it is possible that a higher concentration of the drug would have been more effective in reducing TBI-associated behavioral dysfunction.

Since multiple mechanisms contribute to TBI pathophysiology, it is thought that a drug specific for a single target may not give rise to meaningful improvements in neurobehavioral outcome. There is a general consensus among TBI researchers that either a combination treatment, or a drug that targets multiple TBI-associated pathological cascades, is likely to be effective as a treatment [45]. Based on this premise, we tested the efficacy of lithium, a relatively non-selective inhibitor of GSK-3. Lithium inhibits GSK-3 both directly [36], [46], and indirectly by reducing the activity of protein phosphatase-1 [47] and by increasing the phosphorylation of GSK-3 via activation of Akt [48]. Lithium has been shown to attenuate multiple pathological processes that have been reported to contribute to TBI pathophysiology such as apoptosis, oxidative stress, and mitochondrial and endoplasmic dysfunction. For example, pre-treatment with lithium protects cultured neurons from glutamate-induced cell death [49], [50], and reduces the oxidative stress associated with neuropeptide S [51]. Bipolar patients on lithium have reduced levels of N-acetyl aspartate (NAA), a marker for neural injury, and have more gray matter volume as compared to control patients that are not on lithium therapy [52]–[55]. Although many of these neuroprotective properties of lithium are thought to be due to its influence on GSK-3 [56], lithium has been demonstrated to alter the activity of several other pathways. For example, lithium can indirectly block PKC activity by inhibiting inositol monophosphatase (IMPase) activity [57], thereby reducing the generation of diacylglycerol and inositol triphosphate. Nucleotide bisphosphate 3′-nucleotidase (BPntase, also called RnPIP) is noncompetitively inhibited by lithium [58], whereas lithium has been shown to activate the ERK/MAPK cascade [59]. Although we observed that lithium effectively inhibited GSK-3, we cannot rule out the contribution of these other targets of lithium on the results we observed.

Recently, it has been shown that pretreatment of mice with lithium prior to brain injury reduces inflammation, prevents TBI-induced depression, improves spatial learning and memory, and protects against hippocampal cell loss [25], [60]. Consistent with this, we observed that lithium, when administered post-TBI and continued for an additional 5 days, significantly improved hippocampal function as indicated by improved learning and memory in the Morris water maze task tested days 14–25 post-injury. Stereological cell counts revealed that these functional improvements were accompanied by a significant preservation in CA3 hippocampal neuron numbers. Although studies have shown that cell death after TBI can continue for weeks-to-months, the majority of hippocampal cell loss occurs within the first few days after the injury [8], [61], [62]. As inhibition of GSK-3 has been shown to offer neuroprotection in response to several toxic agents, it is likely that the protection observed was due to a reduction in hippocampal cell death within the initial 5-day period after injury (the time frame of lithium administration). However, we can rule out any lasting neuroprotective effects of lithium treatment that may have contributed to the behavioral and histological improvements we observed.

Although oral lithium carbonate is an effective therapy of treating bipolar disorders, benefit is typically only seen following prolonged use. This delayed onset of action is in contrast to the relatively acute effects described herein. While the mechanism underlying lithium's effect following TBI is not clear, a few studies have suggested that mechanistic differences exist between acute and chronic lithium treatment paradigms. For example, chronic, but not acute, lithium treatment increases BDNF expression in the rat hippocampus and temporal cortex [63], and BDNF expression has been observed following 28days of lithium treatment in persons with bipolar disorder [64]. In contrast, neuroprotection can be observed following acute lithium treatment, an effect thought to be due to a reduction of GSK-3-mediated apoptosis [65]. While we observed that GSK-3 activity could be inhibited using an acute administration routine (daily dosing for 5 days post-injury), a single dose of lithium did not significantly alter GSK-3 phosphorylation or result in significant accumulation of ß-Catenin. Thus, while the treatment window we observed in the present study is relatively shorter than that typically required to treat bipolar disorder, continuous dosing was still required to significantly alter GSK-3 activity.

The therapeutic dose for lithium is in the range of 900 mg of lithium carbonate/day (24 mEq lithium). Usually, these patients receive an initial dose of 300 mg (or 8mEq) to reduce the occurrence of adverse events such as nausea and kidney injury. The dose is then gradually increased to the recommended serum therapeutic dose (0.6–1.2mEq/L). Although we did not monitor the serum level of lithium, previous studies investigating its pharmacokinetics in rats have indicated that 5 mEq/kg lithium reaches a peak serum concentration of approximately 10 mEq/L with a half-life of 7 hr [66]. Thus, we anticipate that 1mEq/kg would give rise to a significantly lower serum concentration that is more comparable to the recommended dose. In addition to treatment of bipolar disorder with lithium alone, combined treatment with lithium and valproic acid has been used to treat patients resistant to monotherapy with either drug [67]. Furthermore, a combination of 60 mg/kg lithium chloride (1.4 mEq) and 300 mg/kg valproic acid delayed disease onset, reduced neurological deficits and prolonged survival in a mouse model of lateral sclerosis as compared to either drug alone [68]. As we have recently shown that valproic acid also offers neuroprotection and improves cognitive function following TBI [69], future studies will determine if a combination of lower doses of lithium (e.g. 0.25 mEq, Figure 2) and valproic acid can be used to reduce the consequences of TBI while minimizing adverse events associated with high doses of either drug [70], [71].

## References

[1] Kochanek PM, Clark RS, Ruppel RA, Adelson PD, Bell MJ (2000). Biochemical, cellular, and molecular mechanisms in the evolution of secondary damage after severe traumatic brain injury in infants and children: Lessons learned from the bedside.. Pediatr Crit Care Med.

[2] Yakovlev AG, Faden AI (1995). Molecular biology of CNS injury.. J Neurotrauma.

[3] Thompson HJ, Lifshitz J, Marklund N, Grady MS, Graham DI (2005). Lateral fluid percussion brain injury: a 15-year review and evaluation.. J Neurotrauma.

[4] Faden AI (2002). Neuroprotection and traumatic brain injury: theoretical option or realistic proposition.. Curr Opin Neurol.

[5] Hayes RL, Wang KK, Kampfl A, Posmantur RM, Newcomb JK (1998). Potential contribution of proteases to neuronal damage.. Drug News Perspect.

[6] Dixon CE, Kochanek PM, Yan HQ, Schiding JK, Griffith RG (1999). One-year study of spatial memory performance, brain morphology, and cholinergic markers after moderate controlled cortical impact in rats.. J Neurotrauma.

[7] Colicos MA, Dixon CE, Dash PK (1996). Delayed, selective neuronal death following experimental cortical impact injury in rats: possible role in memory deficits.. Brain Res.

[8] Yakovlev AG, Knoblach SM, Fan L, Fox GB, Goodnight R (1997). Activation of CPP32-like caspases contributes to neuronal apoptosis and neurological dysfunction after traumatic brain injury.. J Neurosci.

[9] Grimes CA, Jope RS (2001). The multifaceted roles of glycogen synthase kinase 3beta in cellular signaling.. Prog Neurobiol.

[10] Perez M, Rojo AI, Wandosell F, Diaz-Nido J, Avila J (2003). Prion peptide induces neuronal cell death through a pathway involving glycogen synthase kinase 3.. Biochem J.

[11] Watcharasit P, Bijur GN, Zmijewski JW, Song L, Zmijewska A (2002). Direct, activating interaction between glycogen synthase kinase-3beta and p53 after DNA damage.. Proc Natl Acad Sci U S A.

[12] Hu S, Begum AN, Jones MR, Oh MS, Beech WK (2009). GSK3 inhibitors show benefits in an Alzheimer's disease (AD) model of neurodegeneration but adverse effects in control animals.. Neurobiol Dis.

[13] Jope RS, Johnson GV (2004). The glamour and gloom of glycogen synthase kinase-3.. Trends Biochem Sci.

[14] Niehrs C, Shen J (2010). Regulation of Lrp6 phosphorylation.. Cell Mol Life Sci.

[15] Duronio V (2008). The life of a cell: apoptosis regulation by the PI3K/PKB pathway.. Biochem J.

[16] Dixon CE, Clifton GL, Lighthall JW, Yaghmai AA, Hayes RL (1991). A controlled cortical impact model of traumatic brain injury in the rat.. J Neurosci Methods.

[17] Dash PK, Moore AN, Dixon CE (1995). Spatial memory deficits, increased phosphorylation of the transcription factor CREB, and induction of the AP-1 complex following experimental brain injury.. J Neurosci.

[18] Hoskison MM, Moore AN, Hu B, Orsi S, Kobori N (2009). Persistent working memory dysfunction following traumatic brain injury: Evidence for a time-dependent mechanism.. Neuroscience.

[19] Hamm RJ, Dixon CE, Gbadebo DM, Singha AK, Jenkins LW (1992). Cognitive deficits following traumatic brain injury produced by controlled cortical impact.. J Neurotrauma.

[20] Dash PK, Mach SA, Moore AN (2002). The role of extracellular signal-regulated kinase in cognitive and motor deficits following experimental traumatic brain injury.. Neuroscience.

[21] Royo NC, LeBold D, Magge SN, Chen I, Hauspurg A (2007). Neurotrophin-mediated neuroprotection of hippocampal neurons following traumatic brain injury is not associated with acute recovery of hippocampal function.. Neuroscience.

[22] Sullivan PG, Bruce-Keller AJ, Rabchevsky AG, Christakos S, Clair DK (1999). Exacerbation of damage and altered NF-kappaB activation in mice lacking tumor necrosis factor receptors after traumatic brain injury.. J Neurosci.

[23] Coggeshall RE, Lekan HA (1996). Methods for determining numbers of cells and synapses: a case for more uniform standards of review.. J Comp Neurol.

[24] Zhang X, Chen Y, Ikonomovic MD, Nathaniel PD, Kochanek PM (2006). Increased phosphorylation of protein kinase B and related substrates after traumatic brain injury in humans and rats.. J Cereb Blood Flow Metab.

[25] Shapira M, Licht A, Milman A, Pick CG, Shohami E (2007). Role of glycogen synthase kinase-3beta in early depressive behavior induced by mild traumatic brain injury.. Mol Cell Neurosci.

[26] Coghlan MP, Culbert AA, Cross DA, Corcoran SL, Yates JW (2000). Selective small molecule inhibitors of glycogen synthase kinase-3 modulate glycogen metabolism and gene transcription.. Chem Biol.

[27] Ren M, Senatorov VV, Chen RW, Chuang DM (2003). Postinsult treatment with lithium reduces brain damage and facilitates neurological recovery in a rat ischemia/reperfusion model.. Proc Natl Acad Sci U S A.

[28] Miller JS, Tallarida RJ, Unterwald EM (2010). Inhibition of GSK3 attenuates dopamine D1 receptor agonist-induced hyperactivity in mice.. Brain Res Bull.

[29] Colicos MA, Dash PK (1996). Apoptotic morphology of dentate gyrus granule cells following experimental cortical impact injury in rats: possible role in spatial memory deficits.. Brain Res.

[30] Davidson G, Niehrs C (2010). Emerging links between CDK cell cycle regulators and Wnt signaling.. Trends Cell Biol.

[31] Piao S, Lee SH, Kim H, Yum S, Stamos JL (2008). Direct inhibition of GSK3beta by the phosphorylated cytoplasmic domain of LRP6 in Wnt/beta-Catenin signaling.. PLoS One.

[32] Wu G, Huang H, Garcia AJ, He X (2009). Inhibition of GSK3 phosphorylation of beta-Catenin via phosphorylated PPPSPXS motifs of Wnt coreceptor LRP6.. PLoS One.

[33] Fang X, Yu SX, Lu Y, Bast RC, Woodgett JR (2000). Phosphorylation and inactivation of glycogen synthase kinase 3 by protein kinase A. Proc Natl Acad Sci U S A.

[34] Cross DA, Alessi DR, Cohen P, Andjelkovich M, Hemmings BA (1995). Inhibition of glycogen synthase kinase-3 by insulin mediated by protein kinase B.. Nature.

[35] Neary JT, Kang Y (2006). P2 purinergic receptors signal to glycogen synthase kinase-3beta in astrocytes.. J Neurosci Res.

[36] Stambolic V, Ruel L, Woodgett JR (1996). Lithium inhibits glycogen synthase kinase-3 activity and mimics wingless signalling in intact cells.. Curr Biol.

[37] Sutherland C, Leighton IA, Cohen P (1993). Inactivation of glycogen synthase kinase-3 beta by phosphorylation: new kinase connections in insulin and growth-factor signalling.. Biochem J.

[38] Zhang X, Chen Y, Jenkins LW, Kochanek PM, Clark RS (2005). Bench-to-bedside review: Apoptosis/programmed cell death triggered by traumatic brain injury.. Crit Care.

[39] Soutar MP, Kim WY, Williamson R, Peggie M, Hastie CJ (2010). Evidence that glycogen synthase kinase-3 isoforms have distinct substrate preference in the brain.. J Neurochem.

[40] Doble BW, Woodgett JR (2003). GSK-3: tricks of the trade for a multi-tasking kinase.. J Cell Sci.

[41] Beurel E, Jope RS (2006). The paradoxical pro- and anti-apoptotic actions of GSK3 in the intrinsic and extrinsic apoptosis signaling pathways.. Prog Neurobiol.

[42] Facci L, Stevens DA, Skaper SD (2003). Glycogen synthase kinase-3 inhibitors protect central neurons against excitotoxicity.. Neuroreport.

[43] Takadera T, Sakamoto Y, Ohyashiki T (2004). NMDA receptor 2B-selective antagonist ifenprodil-induced apoptosis was prevented by glycogen synthase kinase-3 inhibitors in cultured rat cortical neurons.. Brain Res.

[44] Takadera T, Ohyashiki T (2004). Glycogen synthase kinase-3 inhibitors prevent caspase-dependent apoptosis induced by ethanol in cultured rat cortical neurons.. Eur J Pharmacol.

[45] Margulies S, Hicks R (2009). Combination therapies for traumatic brain injury: prospective considerations.. J Neurotrauma.

[46] Klein PS, Melton DA (1996). A molecular mechanism for the effect of lithium on development.. Proc Natl Acad Sci U S A.

[47] Zhang F, Phiel CJ, Spece L, Gurvich N, Klein PS (2003). Inhibitory phosphorylation of glycogen synthase kinase-3 (GSK-3) in response to lithium. Evidence for autoregulation of GSK-3.. J Biol Chem.

[48] Chalecka-Franaszek E, Chuang DM (1999). Lithium activates the serine/threonine kinase Akt-1 and suppresses glutamate-induced inhibition of Akt-1 activity in neurons.. Proc Natl Acad Sci U S A.

[49] Hashimoto R, Hough C, Nakazawa T, Yamamoto T, Chuang DM (2002). Lithium protection against glutamate excitotoxicity in rat cerebral cortical neurons: involvement of NMDA receptor inhibition possibly by decreasing NR2B tyrosine phosphorylation.. J Neurochem.

[50] Leng Y, Liang MH, Ren M, Marinova Z, Leeds P (2008). Synergistic neuroprotective effects of lithium and valproic acid or other histone deacetylase inhibitors in neurons: roles of glycogen synthase kinase-3 inhibition.. J Neurosci.

[51] Castro AA, Casagrande TS, Moretti M, Constantino L, Petronilho F (2009). Lithium attenuates behavioral and biochemical effects of neuropeptide S in mice.. Peptides.

[52] Moore GJ, Bebchuk JM, Hasanat K, Chen G, Seraji-Bozorgzad N (2000). Lithium increases N-acetyl-aspartate in the human brain: in vivo evidence in support of bcl-2's neurotrophic effects?. Biol Psychiatry.

[53] Moore GJ, Bebchuk JM, Wilds IB, Chen G, Manji HK (2000). Lithium-induced increase in human brain grey matter.. Lancet.

[54] Silverstone PH, Wu RH, O'Donnell T, Ulrich M, Asghar SJ (2003). Chronic treatment with lithium, but not sodium valproate, increases cortical N-acetyl-aspartate concentrations in euthymic bipolar patients.. Int Clin Psychopharmacol.

[55] Drevets WC (2001). Neuroimaging and neuropathological studies of depression: implications for the cognitive-emotional features of mood disorders.. Curr Opin Neurobiol.

[56] Aghdam SY, Barger SW (2007). Glycogen synthase kinase-3 in neurodegeneration and neuroprotection: lessons from lithium.. Curr Alzheimer Res.

[57] Atack JR, Cook SM, Watt AP, Ragan CI (1992). Measurement of lithium-induced changes in mouse inositol(1)phosphate levels in vivo.. J Neurochem.

[58] Spiegelberg BD, Xiong JP, Smith JJ, Gu RF, York JD (1999). Cloning and characterization of a mammalian lithium-sensitive bisphosphate 3′-nucleotidase inhibited by inositol 1,4-bisphosphate.. J Biol Chem.

[59] Yuan PX, Huang LD, Jiang YM, Gutkind JS, Manji HK (2001). The mood stabilizer valproic acid activates mitogen-activated protein kinases and promotes neurite growth.. J Biol Chem.

[60] Zhu ZF, Wang QG, Han BJ, William CP (2010). Neuroprotective effect and cognitive outcome of chronic lithium on traumatic brain injury in mice.. Brain Res Bull.

[61] Clark RS, Kochanek PM, Watkins SC, Chen M, Dixon CE (2000). Caspase-3 mediated neuronal death after traumatic brain injury in rats.. J Neurochem.

[62] Colicos MA, Dash PK (1996). Apoptotic morphology of dentate gyrus granule cells following experimental cortical impact injury in rats: possible role in spatial memory deficits.. Brain Res.

[63] Fukumoto T, Morinobu S, Okamoto Y, Kagaya A, Yamawaki S (2001). Chronic lithium treatment increases the expression of brain-derived neurotrophic factor in the rat brain.. Psychopharmacology (Berl).

[64] de Sousa RT, van de Bilt MT, Diniz BS, Ladeira RB, Portela LV (2011). Lithium increases plasma brain-derived neurotrophic factor in acute bipolar mania: a preliminary 4-week study.. Neurosci Lett.

[65] Mora A, Sabio G, Gonzalez-Polo RA, Cuenda A, Alessi DR (2001). Lithium inhibits caspase 3 activation and dephosphorylation of PKB and GSK3 induced by K+ deprivation in cerebellar granule cells.. J Neurochem.

[66] Wraae O (1978). The pharmacokinetics of lithium in the brain, cerebrospinal fluid and serum of the rat.. Br J Pharmacol.

[67] Lin D, Mok H, Yatham LN (2006). Polytherapy in bipolar disorder.. CNS Drugs.

[68] Feng HL, Leng Y, Ma CH, Zhang J, Ren M (2008). Combined lithium and valproate treatment delays disease onset, reduces neurological deficits and prolongs survival in an amyotrophic lateral sclerosis mouse model.. Neuroscience.

[69] Dash PK, Orsi SA, Zhang M, Grill RJ, Pati S (2010). Valproate administered after traumatic brain injury provides neuroprotection and improves cognitive function in rats.. PLoS One.

[70] Grandjean EM, Aubry JM (2009). Lithium: updated human knowledge using an evidence-based approach: part III: clinical safety.. CNS Drugs.

[71] Haddad PM, Das A, Ashfaq M, Wieck A (2009). A review of valproate in psychiatric practice.. Expert Opin Drug Metab Toxicol.

